# The COVID-19 quandemic

**DOI:** 10.1186/s12992-024-01024-0

**Published:** 2024-03-02

**Authors:** Olivier Rubin, Carina King, Johan von Schreeb, Claudia Morsut, Gyöngyi Kovács, Emmanuel Raju

**Affiliations:** 1 Department of Social Sciences and Business, Roskilde University, Roskilde, Denmark; 2 Department of Global Public Health, Karolinska Institutet, Stockholm, Sweden; 3 Department of Safety, Economics and Planning, Faculty of Science and Technology, University of Stavanger, Stavanger, Norway; 4 HUMLOG Institute, Hanken School of Economics, Helsinki, Finland; 5 Global Health Section and The Copenhagen Centre for Disaster Research, Department of Public Health, University of Copenhagen, Copenhagen, Denmark

**Keywords:** COVID-19, Nordic region, Information, Quantification

## Abstract

**Background:**

The terms syndemic and infodemic have both been applied to the COVID-19 pandemic, and emphasize concurrent socio-cultural dynamics that are distinct from the epidemiological outbreak itself. We argue that the COVID-19 pandemic has exposed yet another important socio-political dynamic that can best be captured by the concept of a quandemic – a portmanteau of “quantification” and “pandemic”.

**Main text:**

The use of quantifiable metrics in policymaking and evaluation has increased throughout the last decades, and is driven by a synergetic relationship between increases in supply and advances in demand for data. In most regards this is a welcome development. However, a quandemic, refers to a situation where a small subset of quantifiable metrics dominate policymaking and the public debate, at the expense of more nuanced and multi-disciplinary discourse. We therefore pose that a quandemic reduces a complex pandemic to a few metrics that present an overly simplified picture. During COVID-19, these metrics were different iterations of case numbers, deaths, hospitalizations, diagnostic tests, bed occupancy rates, the R-number and vaccination coverage. These limited metrics came to constitute *the* internationally recognized benchmarks for effective pandemic management.

Based on experience from the Nordic region, we propose four distinct dynamics that characterize a quandemic: 1) A limited number of metrics tend to dominate both political, expert, and public spheres and exhibit a great deal of rigidity over time. 2) These few metrics crowd-out other forms of evidence relevant to pandemic response. 3) The metrics tend to favour certain outcomes of pandemic management, such as reducing hospitalization rates, while not capturing potential adverse effects such as social isolation and loneliness. 4) Finally, the metrics are easily standardized across countries, and give rise to competitive dynamics based on international comparisons and benchmarking.

**Conclusion:**

A quandemic is not inevitable. While metrics are an indispensable part of evidence-informed policymaking, being attentive to quandemic dynamics also means identifying relevant evidence that might not be captured by these few but dominant metrics. Pandemic responses need to account for and consider multilayered vulnerabilities and risks, including socioeconomic inequities and comorbidities.

## Background

COVID-19 has revitalized the relevance of concepts relying on different morphological derivations of the word “pandemic.” Some have framed the COVID-19 outbreak as a *syndemic*—the synergistic nature of the pandemic based on its interaction with existing global health threats, such as seasonal epidemics and non-communicable diseases, as well as economic and socio-cultural factors including stigmatization, racism, and violence [1, 2]. Conceived in the 1990s, the concept of syndemics captures how these societal, economic and clinical factors shape disease interactions, i.e. co-morbidities and multimorbidity, into producing more severe public health outcomes especially for vulnerable social groups.

Scholars have also emphasized the concept of an *infodemic* during COVID-19. The term was originally coined during the SARS-pandemic in 2003 to describe a global phenomenon of misinformation and rumours that primarily stemmed from alternative news outlets [3]. However, during the COVID-19 pandemic the concept caught on at unprecedented speeds in both academia and practice. Most understandings of infodemics emphasize both the *volume* of information coming from multiple sources, and the misleading or false *nature* of much of that information [4, 5]. Left unmanaged, an infodemic can undermine trust, erode public support for key public health interventions (e.g. vaccination or physical distancing), and elevate risks for civil disobedience, unrest, and anxiety [6]. Therefore, attention to infodemic management, as well as fact-checking, became mainstreamed across both national and international health agencies [5, 7].

The terms syndemic and infodemic both emphasize concurrent socio-cultural dynamics that are distinct from the epidemiological outbreak itself. Syndemics emphasise the intersectional social vulnerabilities of disease outbreaks, whereas infodemics focus on the adverse consequences of having too much (unreliable) information circulating during outbreaks.

Based on our collective experience in the Nordic region, we argue that the COVID-19 pandemic has exposed yet another important socio-political dynamic that can best be captured by the concept of a *quandemic* – a portmanteau of “quantification” and “pandemic”. The use of quantifiable metrics in policymaking and evaluation has increased throughout the last decades [8]. This development is driven by a synergetic relationship between increases in supply and advances in demand for data. Today, we can generate, access and analyse quantifiable metrics for most aspects of our societies, and these metrics are in great demand for evidence-based/informed decision-making approaches [9]. In most regards this is a welcome development. A quandemic, therefore, does not refer to the mere use of quantifiable metrics in policymaking. Rather, it refers to a situation where a small subset of quantifiable metrics dominate policymaking and the public debate. In the Nordic region, we observed a widespread and competitive comparison across the Nordic countries based on these metrics, despite differing policy intentions. We therefore pose that a quandemic reduces a complex pandemic to a few metrics that present an overly simplified picture. During COVID-19, these metrics were different iterations of case numbers, deaths, hospitalizations, diagnostic tests, bed occupancy rates, the R-number and vaccination coverage. In themselves, these metrics are critical indicators of pandemic progression, and were rightly collated by the World Health Organisation at a global level – but they also came to constitute *the* internationally recognized benchmarks for effective pandemic management [10].

## Main text

The quandemic concept has clear roots in Michael Foucault’s notion of biopower [11, 12]. Foucault introduced the concept of biopower to denote state power over populations and individuals that hinges fundamentally on expert knowledge of the population’s biological quality and longevity [13]. Biopower seeks to optimize a population’s vitality mainly through rationalized mechanisms of population monitoring and medicalization [14]. One important expression of and prerequisite for biopower is quantification. In the 1970s, Foucault described how this practice became apparent during a smallpox outbreak in the eighteenth-century. The primary focus was no longer understanding the pathology of the epidemic itself but to track the number of the infected, their age, medical consequences, and mortality using statistical methods. In the words of Foucault: “*when quantitative analyses are made of smallpox in terms of success and failure […] the disease no longer appears in this solid relationship of the prevailing disease to its place or milieu, but as a distribution of cases in a population circumscribed in time or space*” [15]. Since then, numbers and statistics have come to play a crucial role in epidemic and crisis management. However, Foucault reminds us that metrics are not only important pieces of evidence but, simultaneously, they are expressions of biopower. Decisions of what metrics to promote or ignore, and how to measure them have the power to frame the pandemic in a certain political light and therefore shape responses.

We propose four distinct dynamics that characterize a quandemic:

(i) A few metrics tend to dominate both political, expert, and public spheres and they exhibit a great deal of rigidity over time. The metrics are produced and reproduced by key stakeholders within and across the different spheres of influence without much open debate and discussion of alternatives. Instead, the metrics are followed and reported regularly by health agencies, politicians, and major media outlets. In addition, the same metrics dominate throughout the pandemic. While new metrics might emerge, such as vaccination rates, they largely serve to accentuate the importance of the existing metrics.
(ii) These few metrics appear to crowd-out other forms of evidence relevant to pandemic response. These alternative sources of evidence can be qualitative and quantitative in nature and represent socio-economic or public health dynamics. Examples of crucial but deprioritised evidence could include anthropological perspectives of vaccine hesitancy and community engagement, economic approaches to vulnerability, and quantitative tracking of mental health impacts (public health); gender violence (social) or differences in student attainment following prolonged periods of distance teaching (educational). While it is important to emphasize that this evidence was far from ignored during COVID-19, the quantitative metrics would often constitute the point of departure for debates and deliberations and the additional evidence would primarily be an addendum used to contextualize and qualify [16, 17].
(iii) The metrics tend to favour certain outcomes of pandemic management. During COVID-19, non-pharmaceutical interventions would almost certainly improve these metrics (to varying degrees), while the potential adverse impacts of the interventions would not be the focus on these metrics. These adverse consequences would, therefore, need to be considered on an ad-hoc basis. The benefits of lockdowns would be captured by the metrics, e.g. a drop in cases, hospitalizations, and deaths. Whereas the costs of these interventions were largely beyond these dominant metrics. Disaster management studies have long been attentive of the need to address the socio-economic consequences of *both* the hazard itself as well as the mitigating measures [18]. The dominant metrics during COVID-19 appeared ill-equipped to capture the nuanced and longer-term impacts of the pandemic response.
(iv) Finally, the metrics are easily standardized across countries, and give rise to competitive dynamics based on international comparisons and benchmarking. While the metrics during COVID-19 faced limited competition internally from other types of evidence, they exhibited a substantial potential for generating external competition between countries and different administrations. Pandemic successes and failures were evaluated and compared in terms of this limited subset of metrics. Political leaders were faced daily with these metrics and were often held accountable for unfavourable developments compared to other similar countries and over time. This created a textbook suboptimal situation where decision makers would pursue policies that carried concentrated and visible benefits (lowering mortality rates, for example) while keeping the costs dispersed and less visible [19]. Policymaking can be caught in a self-fulfilling loop where the initial focus on these metrics continuously reinforces the political salience of the same metrics.

To be clear, having access to standardized measures on a wide range of health outcomes constitutes best practice during pandemic management. In fact, many countries with limited capacity faced a substantial impediment to effective pandemic management because they had little access to these types of timely and disaggregated national metrics. However, a quandemic concerns the overreliance on these metrics and the resulting unproductive competitive comparisons. We observed these quandemic dynamics in the Nordic region. From the very initial phases of the pandemic, it was clear that a few metrics permeated the political and public debates. Across the Nordic countries, the main newspapers outlets carried the development of these key metrics daily on their frontpage and/or main website. Cases and fatalities came to embody the success of the pandemic response. Only towards the end of the first wave did Finland, for example, assemble a group of scientists that were to monitor COVID-19-related results in a way that paid attention to other factors including education, the economy, to technological innovation, misinformation, and resilience [20]. Sweden famously pursued slightly more lenient non-pharmaceutical interventions in 2020, motivated by the Swedish Health Agency’s emphasis on additional longer-term objectives that were not easily caught in the metrics. Equity was stated as the overarching focus of agency’s mission statement and was highlighted as a key guiding principle by the actors involved in key advice making during COVID-19 [21, 22]. The decision not to close primary schools down for physical attendance nationally, for example, was rooted in a concern for ensuring educational attainment, access to school meals, and the social well-being of children and had full support of the Swedish Children’s Ombudsman – the highest legal authority for the rights of children [22, 23].

This approach was met with scepticism internationally, and to some extent nationally, as the dominant metrics deteriorated in the Autumn and Winter of 2020 compared to other Nordic countries of Denmark, Norway and Finland [24–26]. These numbers became overtly political, with the other Nordic governments using Sweden as a cautionary tale of laissez faire pandemic management. Danish and Norwegian newspapers carried many comparisons to the Swedish strategy equating the success of their pandemic response by the lower rates of cases and deaths in 2020 compared to Sweden [27, 28]. In February 2021, the Danish government emphasized that Denmark only had one fourth of the infected compared to Sweden [29]. The correctness of Sweden's initial pandemic strategy is not the point here. Rather success or failure at the time was primarily assessed by a handful of quantitative metrics that did not reflect the national pandemic response goals. Therefore, debating achievements based only on these metrics risks obscuring comprehension.

Three years later, the media and politicians engaged rigorously in yet another comparison of pandemic responses across the Nordic countries. This time the comparison was based on excess mortality rates during the pandemic and was reported in the Norwegian media, [30] Danish media, [31] Finnish media, [32] and the Swedish media [33]. The various measures of excess mortality suggested that Sweden fared well compared to the other Nordic countries, when using population-adjusted excess mortality rates for 2020–2022. While the new excess mortality metric was used to vindicate parts of the Swedish pandemic strategy, the point here is much broader: that such comparison still reinforces the same quandemic mindset that had been dominant in the early phases of the pandemic: a competition of biopower where successes or failures are reduced to a few select metrics. Even excess mortality rates are insufficient to fully gauge the impact of the pandemic as well as the policies implemented to combat it. It leaves out important aspects such as morbidity, the impact on education, equity, economy, mental health, and general wellbeing.

Thus, we propose that the four quandemic characteristics risk producing suboptimal conditions for balanced public debate and policymaking, as evidenced by the Nordic example. A quandemic obscures important syndemic dynamics, as more diverse scientific evidence capturing socio-economic vulnerabilities of the outbreak tends to be muffled by the few dominant metrics. Further, it increases exposure to infodemic dynamics because the dominance of these metrics might create an information void in spaces which they do not capture. Misinformation, pseudo-science, and scientific polarisation can roam in areas where these metrics fall short.

## Conclusion

A quandemic is not inevitable. While metrics are an indispensable part of evidence-informed policymaking, being attentive to quandemic dynamics also means identifying relevant evidence that might not be captured by these few but dominant metrics. Pandemic responses need to account for and consider multilayered vulnerabilities and risks, including socioeconomic inequities and comorbidities. Giving a voice to that type of evidence in the policymaking process appears crucial for an effective response to the next pandemic.

## Data Availability

Not applicable.
